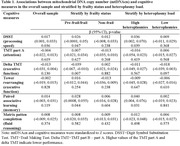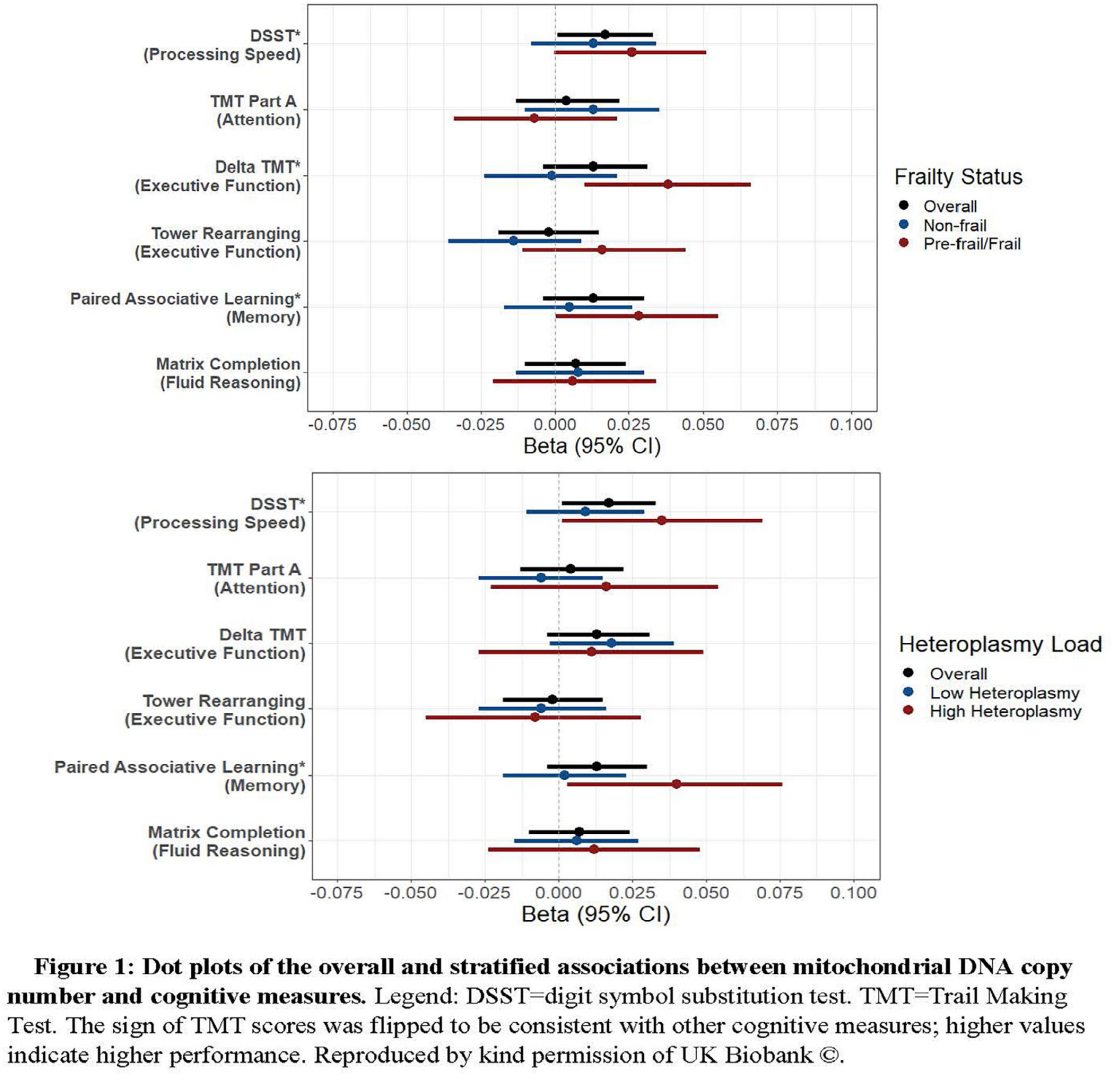# Mitochondrial DNA copy number from human blood is associated with specific cognitive function

**DOI:** 10.1002/alz.087418

**Published:** 2025-01-09

**Authors:** Qu Tian, David A. Zweibaum, Richard Oppong, Yong Qian, Luke C Pilling, Francesco Casanova, Janice L Atkins, David Melzer, Jun Ding, Luigi Ferrucci

**Affiliations:** ^1^ National Institute on Aging, Baltimore, MD USA; ^2^ University of Exeter, Exeter, Devon UK; ^3^ Translational Gerontology Branch, National Institute on Aging, NIH, Baltimore, MD USA

## Abstract

**Background:**

The mitochondrial cascade hypothesis suggests that mitochondrial dysfunction plays an important role in the pathogenesis of Alzheimer’s disease dementia. Recent data have shown that mitochondrial DNA copy number (mtDNAcn) in human blood is associated with dementia risk and cognitive function, but which specific cognitive measures or domains are associated with mitochondrial dysfunction and whether this relationship is affected by health deterioration such as physical frailty or mitochondrial somatic mutations is not clear.

**Methods:**

We measured mtDNAcn and heteroplasmies using fastMitoCalc and MitoCaller, respectively, from UK Biobank Whole Genome Sequencing (WGS) data at study entry (2006‐2010). Pre‐frail/frail status was determined by the presence of slow gait, weight loss, low grip strength, exhaustion, or low physical activity. Cognitive function was assessed in a subset of participants (mean age=64.1,51.7% women, 97.0% White) on average 8.9 years after study entry, including processing speed via Digit Symbol Substitution Test (DSST)(n=13,940), attention via Trail Making Test (TMT) part A(n=13,482), executive function via delta TMT(n=13,428) and tower rearranging task(n=13,819), memory via paired associative learning task(n=14,094), and fluid reasoning via matrix pattern completion task(n=13,935). We examined the associations between mtDNAcn and cognitive measures using multivariate linear regression, adjusted for demographic factors, smoking status, follow‐up time, measures related to mtDNAcn assessment, and Apolipoprotein E ε4 status. We then stratified the analysis by frailty status and levels of heteroplasmy load.

**Results:**

Overall, more mtDNAcn was significantly associated with higher DSST (p=0.036) but not with other cognitive measures. After stratification by frailty status, mtDNAcn was associated with DSST, delta TMT, and paired associative learning in the pre‐frail/frail participants only (p=0.047,0.007, and 0.044, respectively). After stratification by a median split of heteroplasmy load, mtDNAcn was associated with DSST and paired associative learning in those with higher heteroplasmy load (p=0.039 and 0.031, respectively). Results remained similar after excluding participants with dementia diagnosis (n=15).

**Conclusions:**

Higher mitochondrial DNA copy number from human blood is associated with higher cognition in specific measures. The associations with processing speed, executive function, and memory are prominent in individuals with health deterioration of physical frailty or high level of mtDNA heteroplasmy. Future studies are warranted to understand the biological underpinnings.